# Book Review

**Published:** 1917-04

**Authors:** 


**Common Disorders and Diseases of Childhood.** By GEORGE FREDERIC STILL, M.A., M.D. Cantab., F R.C.P. Lond. Third Edition. Pp. xiii., 845. Henry Frowde, Oxford University Press. 1915. 16s. net.—We welcome a third edition of this work, which has, as the author states in his preface, been produced under difficulties in this time of war. In spite of these difficulties, however, Dr. Still has added a chapter on that little-understood disorder of childhood, "Cœliac Disease," and another on "Tuberculous Disease of the Cervical Glands." There has also been a revision of the other chapters, which now embody the latest advances in medical knowledge. The great value of the book, however, is that it is not only the outcome of the author's own vast clinical experience in meeting the many difficulties with which the practitioner is confronted in the daily routine of his practice when dealing with the common disorders of sick children, but includes also valuable criticism of the most recent work of other observers in the same field of medical science.

**Urgent Surgery.** By FELIX LEJARS, translated by W. S. DICKIE, F.R.C.S., and E. WARD, M.A., M.D., F.R.C.S. Vol. II. Third English impression. Pp. ix., 588. Bristol: J. Wright and Sons Ltd. 1915. Price 25s. net.—We have pleasure in commending the second volume of Lejars's original book, which is profusely illustrated, and its production is a credit to the author and to the publishers. Reverdin's needle appears to be in very general use. We observe that a circular wire suture seems to be preferred for fractured patella, although other varieties of suture are most commonly employed by British surgeons. There is a particularly useful chapter on "Injuries of Tendons," with numerous figures explanatory of the varieties of useful sutures which may be employed in these injuries. The volume comprises the consideration of injuries of the genito-urinary organs, the rectum and extremities, together with a section on hernia, and we welcome its appearance and congratulate the author and his collaborators.

